# Direct Observation of Hydrangea Blue-Complex Composed of 3-*O*-Glucosyldelphinidin, Al^3+^ and 5-*O*-Acylquinic Acid by ESI-Mass Spectrometry

**DOI:** 10.3390/molecules23061424

**Published:** 2018-06-12

**Authors:** Takaaki Ito, Kin-ichi Oyama, Kumi Yoshida

**Affiliations:** 1 Graduate School of Information Sciences, Nagoya University, Chikusa, Nagoya 464-8601, Japan; ito.takaaki@b.mbox.nagoya-u.ac.jp; 2 Research Institute for Materials Science, Nagoya University, Chikusa, Nagoya 464-8602, Japan; oyama@cic.nagoya-u.ac.jp; 3 Graduate School of Informatics, Nagoya University, Chikusa, Nagoya 464-8601, Japan

**Keywords:** Aluminum ion, blue color development, 5-*O*-caffeoylquinic acid, 3-*O*-glucosyldelphinidin, *Hydrangea macrophylla*, ESI-mass, metal complex

## Abstract

The blue sepal color of hydrangea is due to a metal complex anthocyanin composed of 3-*O*-glucosyldelphinidin (**1**) and an aluminum ion with the co-pigments 5-*O*-caffeoylquinic acid (**2**) and/or 5-*O*-*p*-coumaroylquinic acid (**3**). The three components, namely anthocyanin, Al^3+^ and 5-*O*-acylquinic acids, are essential for blue color development, but the complex is unstable and only exists in an aqueous solution. Furthermore, the complex did not give analyzable NMR spectra or crystals. Therefore, many trials to determine the detailed chemical structure of the hydrangea-blue complex have not been successful to date. Instead, via experiments mixing **1**, Al^3+^ and **2** or **3** in a buffered solution at pH 4.0, we obtained the same blue solution derived from the sepals. However, the ratio was not stoichiometric but fluctuated. To determine the composition of the complex, we tried direct observation of the molecular ion of the complex using electrospray-ionization mass spectrometry. In a very low-concentration buffer solution (2.0 mM) at pH 4.0, we reproduced the hydrangea-blue color by mixing **1**, **2** and Al^3+^ in ratios of 1:1:1, 1:2:1 and 1:3:1. All solution gave the same molecular ion peak at *m*/*z* = 843, indicating that the blue solution has a ratio of 1:1:1 for the complex. By using **3**, the observed mass number was *m*/*z* = 827 and the ratio of **1**, **3** and Al^3+^ was also 1:1:1. A mixture of **1**, 3-*O*-caffeoylquinic acid (**4**) and Al^3+^ did not give any blue color but instead was purple, and the intensity of the molecular ion peak at *m*/*z* = 843 was very low. These results strongly indicate that the hydrangea blue-complex is composed of a ratio of 1:1:1 for **1**, Al^3+^ and **2** or **3**.

## 1. Introduction

**Scheme 1:**
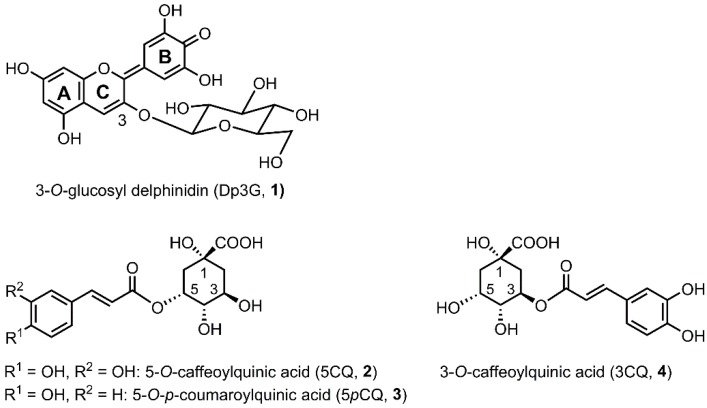
Structures of pigment and co-pigments in hydrangea sepal.

Hydrangea (*Hydrangea macrophylla*) originated from Japan, and what we consider to be its flower is not a true flower but a sepal. Its original sepal color is blue and very famous for its changes in hue with soil conditions, from red through purple to blue [1,2]. In the early 20th century, it was already known that hydrangea cultivated in acidic soil is blue [3,4]. In this condition, aluminum ion (Al^3+^) in soil becomes water-soluble and is absorbed from roots, followed by transport to sepal vacuoles to give a blue color [1,5,6]. In the mid-20th century, the sepal components were reported: colored sepals contained one anthocyanin component, 3-*O*-glucosyldelphinidin (Dp3G, **1**), and three co-pigment components: 5-*O*-caffeoyl-quinic acid (5CQ, **2**), 5-*O*-*p*-coumaroylquinic acid (5*p*CQ, **3**) and 3-*O*-caffeoylquinic acid (3CQ, **4**). These compounds can develop all of the reported sepal colors, red, purple and blue (Scheme 1) [5,7,8,9,10]. In the late 20th century, the different effects of the three co-pigments on blue coloration were reported [11]. The 5-*O*-acylquinic acids (**2** and **3**) and Al^3+^ were revealed to be essential for blue coloration [11]. However, further investigation into the chemical structure and mechanism of blue color development was not performed.

We are interested in this phenomenon and have tried to reveal the chemical mechanisms of the color variation of hydrangea [12,13,14,15,16]. In 2003, we measured the vacuolar pH of colored sepal cells using the microelectrode method and reported that the pHv of the blue cells was 4.1, which is significantly higher than that of the red cells (pHv: 3.3) [12]. We next synthesized various natural and unnatural co-pigment derivatives and performed reproduction experiments by mixing **1**, co-pigments and Al^3+^. As a result, the essential structure of co-pigment for blue coloration was clarified as the 1-OH, 1-COOH, 5-ester structure in quinic acid, and the aromatic acyl moiety had a stabilizing effect through a hydrophobic interaction with the anthocyanidin chromophore [13,14]. In 2009, quantitative analysis of **1**–**4** and Al^3+^ in blue and red sepal cells was performed, and it was clarified that the concentration of **1** in blue and red cells was not very different, but the molar ratio of **2** and **3** and Al^3+^ in blue cells was much higher than that of red cells [15]. However, neither the structure nor the ratio of the components in the blue colored pigment in hydrangea could be determined because the blue pigment can exist only in aqueous solutions and did not give an analyzable NMR spectrum.

We named the complex that develops blue color in hydrangea in sepals as “hydrangea blue-complex” and have performed experiments to elucidate its composition and chemical structure. Recently, we measured the ^1^H-NMR spectrum of the hydrangea blue-complex by mixing **1**, **2** and Al^3+^ in a ratio of 1:2:1 in 6 M acetate buffer at pD 4.0 [17]. Analysis of the spectrum gave a partial structural information, indicating that in the hydrangea-blue complex, Dp3G (**1**) might exist in an equilibrium between chelating and non-chelating structures that have an interaction with 5CQ (**2**). Therefore, we proposed a schematic structure of the hydrangea blue-complex as a 1:1:1 complex of **1**, **2** and Al^3+^ [17]. However, the composition was not determined unambiguously.

For the mass analysis of such unstable metal complex anthocyanins, electrospray ionization is a very powerful tool. We determined the molecular weight of several metalloanthocyanins [16] such as commelinin [18], protocyanin [19], protodelphin [20] and cyanosalvianin [21]. Therefore, we applied this ionization method for determination of the composition of hydrangea blue-complex. In this study, we reproduced the blue color of hydrangea in very low concentrated buffer solution (2 mM) at pH 4.0, and the blue solution was analyzed using ESI-TOF Mass. We detected the molecular ion peak of hydrangea blue-complex for the first time.

## 2. Results and Discussion

### 2.1. Reproduction of Blue and Red Sepal Colors In Vitro

As previously reported [13,14,17], we performed reproduction experiments by mixing Dp3G (**1**), co-pigment and Al^3+^ in various buffer solutions at a concentration of 100 mM. For NMR measurements, we used an unusually concentrated buffer such as 6 M [17]. In both experiments, we obtained the same blue solution, with identical Vis and CD spectra to the hydrangea sepals and protoplast [13,14,15]. However, in ESI-Mass analysis with such a high concentrated salt solution, the molecular ion peak of the complex was hardly detected; only the molecular ion peaks attributable to the monomer anthocyanin and co-pigment were observed. This phenomenon, inhibition of ionization by co-existing salts, is usually observed in mass spectrometry. Therefore, we tried to reproduce the hydrangea blue-complex in very low mM salt solutions. Using preliminary experiments, it was found that the blue solution in an acetate buffer less than 5 mM gave a molecular ion peak for the hydrangea blue-complex, and therefore, we chose the concentration of the buffer as 2 mM.

**Figure 1:**
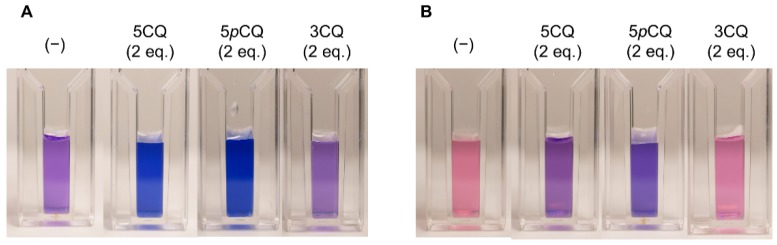
Color of reproduced solutions via mixing of Dp3G (**1**, 0.1 mM) and Al^3+^ (0.1 mM, 1 eq. to **1**) with 0.2 mM (2 eq. to **1**) of co-pigment, 5CQ (**2**), 5*p*CQ (**3**), or 3CQ (**4**) in buffered solutions of 2 mM. (**A**) Solutions mixed at pH 4.0. (**B**) Solutions mixed at pH 3.2.

Figure 1 shows the photos of reproduction experiments that mixed **1** (0.1 mM) and 1 eq. of Al^3+^ with 2 eq. of co-pigment (**2**–**4**) in buffer solutions at pH 4.0 (Panel A) and pH 3.2 (Panel B). At pH 4.0, a mixture of **1** and Al^3+^ without co-pigment exhibited a purple color, but the addition of 5CQ (**2**) or 5*p*CQ (**3**) gave blue solutions (Figure 1A). Contrasting with 5-*O*-acylquinic acids, 3CQ (**4**) gave a purple solution (Figure 1A). At pH 3.2, the solution of **1** with 1 eq. of Al^3+^ exhibited a red color. Two equivalents of 5CQ (**2**) gave a purple solution, and the addition of 2 eq. of 5*p*CQ (**3**) color led to a solution that became bluer than that of 5CQ. The solution with 3CQ showed a red solution similar to that without co-pigment (Figure 1B). Those results were the same as in our previous reproduction experiment in buffers with higher concentration.

**Figure 2:**
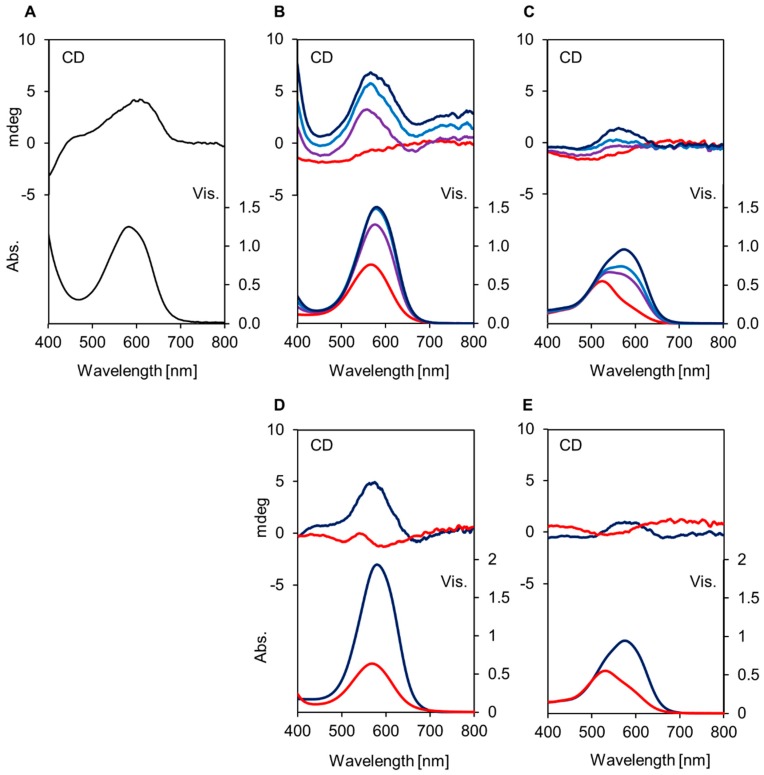
Visible and CD spectra of reproduced solutions by mixing **1** (Dp3G, 0.1 mM) and Al^3+^ (1 eq.) with 0-3 eq. of co-pigment, 5CQ (**2**), 5*p*CQ (**3**), or 3CQ (**4**) in buffered solutions of 2 mM. (**A**) Cell sap obtained from blue hydrangea sepals. (**B**) With 5CQ (**2**) at pH 4.0. —: 0 eq., —: 1 eq., —: 2 eq., —: 3 eq. (**C**) With 5CQ (**2**) at pH 3.2. —: 0 eq., —: 1 eq., —: 2 eq., —: 3 eq. (**D**) With 2 eq. of co-pigment, pH 4.0, —: 5*p*CQ (**3**), —: 3CQ (**4**). (**E**) With 2 eq. of co-pigment, pH 3.2, —: 5*p*CQ (**3**), —: 3CQ (**4**).

Before reproduction experiments, we prepared the pressed juice of blue hydrangea sepals to compare the Vis and CD spectra of the reproduced solutions. Frozen blue sepals were crushed, then, the residues were centrifuged, and the supernatant was filtered to obtain the blue cell sap. Figure 2A shows Vis and CD spectra for the cell sap. In the Vis absorption spectrum, the λ_vismax_ was observed at 580 nm, and in CD, positive Cotton effects were observed near 600 nm, identical to our previous data [13,14,15]. Figure 2B–E show Vis and CD spectra of reproduced solutions by mixing **1** (0.1 mM) and 1 eq. of Al^3+^ with 0 to 3 eq. of co-pigment at pH 4.0 and 3.2, and the data are summarized in Table 1. As shown in Figure 2B, without a co-pigment, the mixture of **1** and Al^3+^ (1 eq.) gave a reddish-purple solution with λ_vismax_ at 566 nm. Upon addition of 5CQ, the solution color became deeper and bluer; the λ_vismax_ of the Vis spectrum shifted to 576 nm (1 eq.), 579 nm (2 eq.) and 581 nm (3 eq.), but the absorbance peaked with 2 eq. of 5CQ. In CD, when 5CQ was added, the positive Cotton effect near 600 nm appeared. These results indicated that the equivalent of co-pigments should affect λ_vismax_ and absorbance. However, it is difficult to determine the composition of the blue complex by only UV-Vis and CD spectral experiments. The color was also affected by the concentration of pigments even if the ratio of the components was the same [13,14,15,17]. We though that this was because the blue complex is unstable in aqueous solutions and exist under equilibrium. Figure 2C shows the results of reproduction experiments in more acidic conditions, at pH 3.2. At pH 3.2, the mixture of **1** and Al^3+^ (1 eq.) gave a red solution with λ_vismax_ at 525 nm. The addition of 5CQ showed hyperchromic and bathochromic effects, but the λ_vismax_ was 543 nm (1 eq.), 567 nm (2 eq.) and 576 nm (3 eq.). Positive Cotton effects near 600 nm were small compared to those at pH 4.0.

Figure 2D,E show the Vis and CD spectra of the reproduced solution obtained by the addition of 2 eq. of 5*p*CQ (**3**) and 3CQ (**4**) at pH 4.0 or 3.2, respectively. At pH 4.0, 2 eq. of 5*p*CQ gave a blue solution with λ_vismax_ at 580 nm, but 3CQ (**4**) gave a reddish-purple-colored solution with λ_vismax_ at 569 nm (Table 1, Figure S1). A positive Cotton effect near 600 nm also indicated that 5*p*CQ gave a similar blue complex to that of 5CQ. At pH 3.2, the λ_vismax_ of the solution of 5*p*CQ was 575 nm and that of 3CQ was 530 nm. All results were identical to our previously reported data in which the hydrangea blue complex could be reproduced by mixing Dp3G (**1**), approximately 2 eq. of 5-*O*-acylquinic acid, and 1 eq. of Al^3+^ at pH 4.0 [13,14,15]. The hydrangea red color was reproduced by mixing Dp3G (**1**) and > 1 eq. 3CQ (**4**) w/wo Al^3+^ at pH 3.2 (Figure 2E, Table 1). Therefore, it was confirmed that even in very low-concentration buffer solutions, the blue and red colors of the hydrangea sepals could be reproduced. Comparing the color of the solution mixing with 2 eq. of co-pigment (5CQ or 5*p*CQ) to Dp3G (**1**, 0.1 mM) and Al^3+^ (1 eq.), 5*p*CQ (**3**) showed a bluer color at 575 nm than that of 5CQ (**2**) with λ_vismax_ at 567 nm (Table 1). By addition of 3 eq. of co-pigment, 5CQ and 5*p*CQ gave almost similar color, but the absorbance was much higher with 5*p*CQ than 5CQ (Table 1, Figure S1). This was because the catechol structure of the caffeoyl residue in 5CQ easily chelates to Al^3+^ to form a 5CQ-Al^3+^ complex and reduces the effective concentration of Al^3+^, which can chelate to form the hydrangea blue–complex. In contrast, 5*p*CQ has no such structure, and therefore, the amount of free Al^3+^ may not decrease. Our previous experiments support this phenomenon. As shown in Figure S2, the addition of Al^3+^ to 5CQ (**2**) showed bathochromic shift of λ_vismax_, but, no such color change was observed in the case of 5*p*CQ (**3**). Furthermore, the chelating position of 5-*O*-acylquinic acid to Al^3+^ in the hydrangea blue-complex is presumed to be 1-COOH, 1-OH and 5-ester [13,14]. At pH 3.2, the 1-COOH may be protonated, and therefore, the chelating efficiency of 1-COOH might be lowered and the dissociation of the blue complex might be promoted. In both pH, pH 4.0 and pH 3.2, 3CQ did not showed typical color change according to increase in co-pigment amount (Table 1). At pH 4.0 3CQ gave a purple colored solution with λ_vismax_ at 569 nm and at pH 3.2 it showed a red color with λ_vismax_ at 531 nm.

### 2.2. ESI-Mass Analysis of Reproduced Blue and Red Solutions

**Figure 3:**
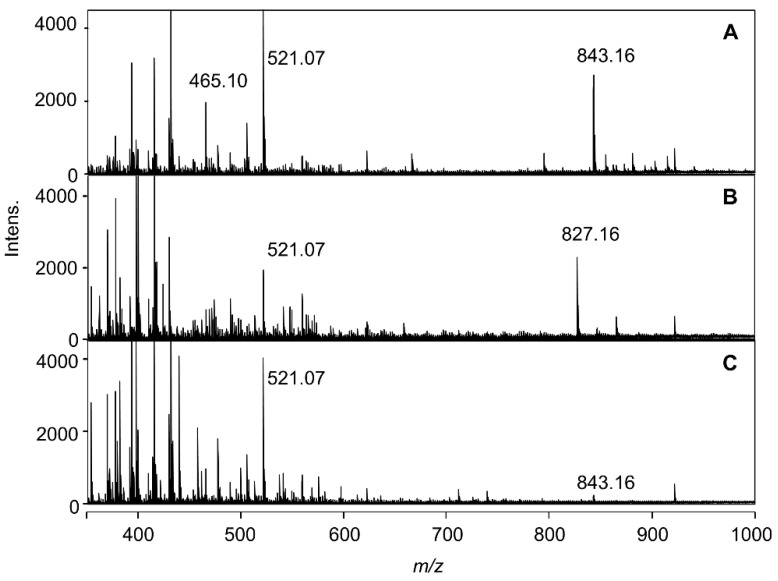
Positive detection ESI-TOF-Mass spectra of reproduced solutions formed by mixing **1** (Dp3G, 0.1 mM) and Al^3+^ (1 eq.) with 2 eq. of co-pigment, 5CQ (**2**), 5*p*CQ (**3**), or 3CQ (**4**) in buffered solutions at pH 4.0 (2 mM). (**A**) 5 CQ (**2**). (**B**) 5*p* CQ (**3**). (**C**) 3 CQ (**4**).

Using the reproduced blue solution in very low-concentration buffered solution (2 mM), the ESI-TOF-Mass spectra were measured. In Figure 3, the mass spectra of the solutions mixed with Dp3G (**1**, 0.1 mM), co-pigment (2 eq.) and Al^3+^ (1 eq.) at pH 4.0 are shown. With positive-ion mode detection, the blue solution mixed with 5CQ gave a molecular ion peak at *m*/*z* = 843.16 (Figure 3A). With negative-ion mode detection, the same solution gave a molecular ion peak at *m*/*z* = 841.14 (Figure S3A). Using high-resolution mass analysis (HR-MS), the molecular formula was determined to be C_37_H_35_O_21_Al (Figure S4A,B), suggesting that the ion is composed of 1:1:1 of Dp3G (**1**), 5CQ (**2**) and Al. When a blue solution obtained by the addition of 5*p*CQ (**3**) was measured, the molecular ion peak was shifted to *m*/*z* = 827.16 (Figure 3B), and with negative-mode detection, the molecular ion was observed at *m*/*z* = 825.14 (Figure S3B). HR-MS analysis gave the molecular formula as C_37_H_35_O_20_Al (Figure S4C,D). However, the reddish-purple solution obtained by 3CQ (**4**) gave a very small peak at *m*/*z* = 843.16 (Figure 3C). All three spectra showed an ion peak at *m*/*z* = 521.07 attributable to Dp3G (**1**, Figure 3). In the buffered solutions and/or during ionization, formation of pseudobase from Dp3G (**1**) by hydration followed by addition of potassium ion gave a pseudobase-potassium ion at *m*/*z* = 521.07 (calcd for C_21_H_22_KO_13_ [Dp3G + OH + K]^+^ 521.07). Potassium ion is known to be easily contaminated from calibration solution of pH meter.

To confirm the composition of each molecular ion peak obtained by mixing Dp3G, 5CQ or 5*p*CQ, and Al^3+^, MS/MS analysis was performed (Figure S5). With positive-mode detection from the molecular ion peak at *m*/*z* = 843.16 (with 5CQ), an ion peak at *m*/*z* = 465.10 attributable to Dp3G (**1**), Al-complex with Dp3G (*m*/*z* = 489.06) and deglucosyl ion (*m*/*z* = 681.11) were observed (Figure S5A). With negative-mode detection from the ion of m/z = 841.15, an ion peak at *m*/*z* = 391.05 attributable to potassium adduct of 5CQ was observed (Figure S5B). The MS/MS analysis from the ion at *m*/*z* = 827.17 (with 5*p*CQ) also gave an ion peak at *m*/*z* = 465.10 attributable to Dp3G (**1**), and from the ion at *m*/*z* = 825.15, an ion peak at *m*/*z* =375.05 and potassium adduct of 5*p*CQ (**3**) were observed (Figure S5C,D). These results strongly suggested that in the blue solution representative of hydrangea blue sepal color, a complex composed of Dp3G, 5-*O*-acylquinic acid and Al^3+^ with the ratio of 1:1:1 should exist, even though the ratio of co-pigment in reproduced solution was 2 eq. to anthocyanin.

**Figure 4:**
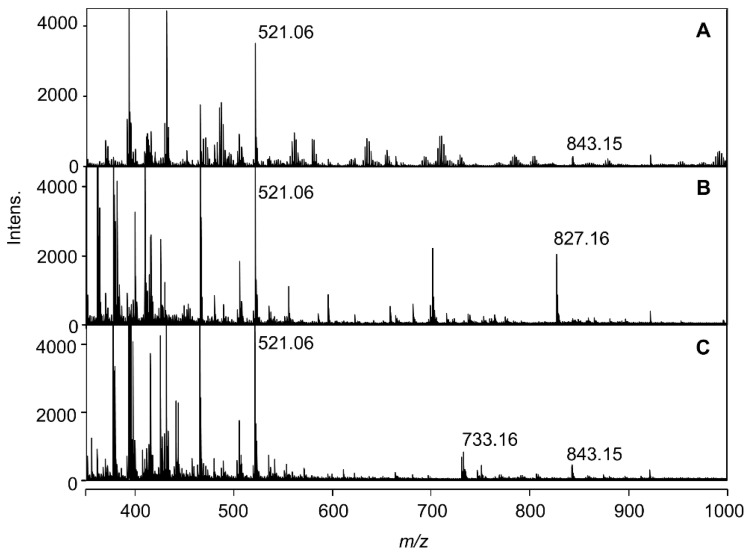
Positive detection ESI-TOF-Mass spectra of reproduced solutions formed by mixing **1** (Dp3G, 0.1 mM) and Al^3+^ (1 eq.) with 2 eq. of co-pigment, 5CQ (**2**), 5*p*CQ (**3**), or 3CQ (**4**) in buffered solutions at pH 3.2 (2 mM). (**A**) 5CQ (**2**). (**B**) 5*p*CQ (**3**). (**C**) 3CQ (**4**).

In Figure 4, the mass spectra of the solutions mixing at pH 3.2 are shown. The 5CQ and 3CQ did not give an obvious molecular ion peak at *m*/*z* = 843.15, but the solution with 5*p*CQ (2 eq.) gave a molecular ion peak at *m*/*z* = 827.16. The molecular formula of this ion peak was confirmed to be C_37_H_36_O_20_Al [M + H]^+^, the same peak detected in Figure 3B. This might give evidence that at pH 3.2, some amount of blue-complex composed of Dp3G, 5*p*CQ and Al^3+^ at a ratio of 1:1:1 exists, though other co-pigments, such as 5CQ and 3CQ, did not produce the blue-colored complex in such conditions, as suggested from the Vis and CD spectra shown in Figure 2.

We also measured Mass using other reproduced blue solutions with different equivalents of 5CQ (1, 3 eq. to **1**) at pH 4.0 (Figure S6). In the mass spectra, the molecular ion peak at *m*/*z* = 843.16 was observed. During all the mass experiments with the mass range to 3500, no ion peak which was composed of Dp3G-5CQ-Al = 1:2:1 and 1:3:1, was detected. These results strongly suggested that in blue solutions reproduced with any ratio of the three components **1**, **2** and Al^3+^, the complex composed of a ratio of 1:1:1 forms. As mentioned in 2.1, using Vis and CD experiments we could not determine the ratio of complex because the increase in equivalent of co-pigments to **1** increased absorbance at λ_vismax_. This was because hydrangea blue-complex in buffered solutions was more unstable than other stoichiometric metal complex pigments such as commelinin and protocyanin [16]. This instability gave the characteristics of hydrangea sepal color being easy to change.

### 2.3. Chemical Structure of the Hydrangea Blue-Complex

**Scheme 2:**
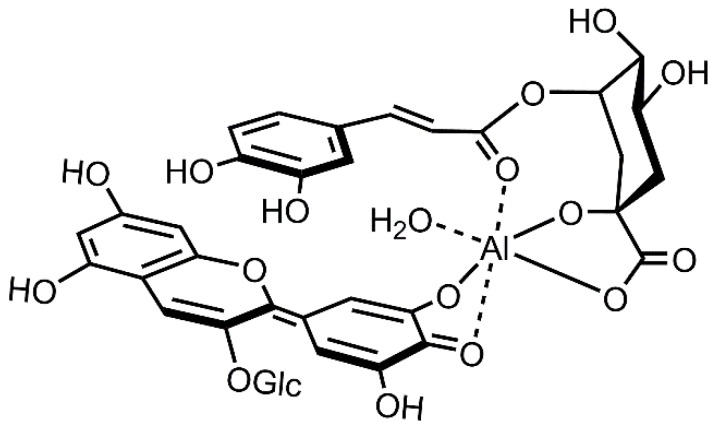
Structure of hydrangea blue-complex.

Unfortunately, the direct mass analysis of cell sap did not give the ion, due to the high salt concentration; the conc. of K^+^ was higher than 30 mM with ICP-AES analysis (Table S1). However, the results of ESI-TOF-Mass analysis of the reproduced blue-colored solution in a very low-concentration buffer solution gave a distinct molecular ion peak of the complex at *m*/*z* = 843 with 5CQ and at 827 with 5*p*CQ. This should give strong evidence that the hydrangea blue-complex was composed of Dp3G (**1**), 5CQ (**2**) or 5*p*CQ (**3**) and Al^3+^ in a 1:1:1 ratio. Combined with our previous reproduction experiment using unnatural co-pigments and NMR analysis in 6 M buffer [17], we confirmed the structure of the blue complex shown in Scheme 2. Al^3+^ chelates to the catechol at the B-ring of Dp3G (**1**) to produce quinonoidalbase anion in weakly acidic media, and the hydrangea blue color was developed. In addition, Al^3+^ chelates to the 1-COOH, 1-OH and 5-ester residues of co-pigments, 5CQ (**2**) and 5*p*CQ (**3**) [13,14]. This complex with six-coordination should give a relatively stable coordination structure of Al^3+^ and also stabilizes the anthocyanidin chromophore by hydrophobic interactions between aromatic acyl group of quinic acid ester. However, the coordination stability might not be so high, and thus, the hydrangea blue-complex may exist in equilibrium between the coordination and dissociation states of co-pigments. Therefore, the NMR spectrum of the blue solution may give both broad and unanalyzable signals of components in complex with sharp signals derived from free 5CQ.

## 3. Materials and Methods

### 3.1. Plant Materials

*H. macrophylla* cv. Narumi blue was donated by Okumura Farm (Toyoake, Aichi) and cultivated in the Botanical Garden, Nagoya University Museum.

### 3.2. Chemicals and Reagents

Dp3G (**1**) was purified from the seed coat of the scarlet bean, *Phaseolus coccineus*, according to our procedure [17]. The co-pigments 5CQ (**2**) and 5*p*CQ (**3**) were synthesized [17,22], and 3CQ (**2**) was purchased from WAKO. Formate buffer (pH 3.2) was prepared by mixing 5 mM formic acid (diluting of 88% Formic acid, WAKO, Osaka, Japan) and 5 mM sodium formate (dissolving of sodium formate, WAKO). Acetate buffer (pH 4.0) was prepared by mixing 5 mM acetic acid (diluting of 99% acetic acid, WAKO) and 5 mM sodium acetate (dissolving of sodium acetate, WAKO). Then, 1 mM aluminum solutions were prepared with AlCl_3_·12H_2_O (Kanto Kagaku, Tokyo, Japan) by dissolution in distilled water.

### 3.3. Cell Sap Preparation

Blue hydrangea sepals were frozen with liquid nitrogen in a mortar, crushed with a pestle, and centrifuged (4 °C, 48,000× *g*, 30 min) in a tube with himac CR21F (Hitachi, Tokyo, Japan). After centrifugation, the supernatant (cell sap) was collected by filtering with a cellulose acetate filter (0.45 μM, TOYO Roshi, Tokyo, Japan).

### 3.4. Reproduction of Hydrangea Blue Color

Stock solutions of Dp3G (**1**, 1 mM) and co-pigments 5CQ (**2**, 2 mM), 5*p*CQ (**3**, 2 mM), and 3CQ (**4**, 2 mM) were prepared just before reproduction experiments by dissolution in water. To a microtube with the volume of 1.5 mL, stock solutions of **1** (70 μL, 100 μM), aluminum solution (70–210 μL, 1–3 eq. to **1**), co-pigment (0–105 μL 0–3 eq.) and a buffer solution (2 mM, 280 μL) were added, and water was added to the mixture to a final solution volume of 700 μL. The solution was mixed, and the pH was measured with a D-21 pH meter (Horiba, Kyoto, Japan) and a LAQUA 9618S-10D electrode (Horiba).

### 3.5. Measurement of Vis and CD Spectra

Visible adsorption spectra (Vis) were recorded with a UV V-550 spectrometer (Jasco, Hachioji, Japan) from 400 to 800 nm with a scanning rate of 400 nm min^−1^ at 25 °C. CD spectra were measured with a CD J-720 spectrometer (Jasco) over the range of 400–800 nm with a scanning rate of 500 nm min^−1^ at 25 °C, and the mean of 4 trials was determined. The spectra of cell sap were measured in a quartz cell with a path length of 0.1 mm, and those of the reproduced solutions were tested in a quartz cell with a 10-mm path length.

### 3.6. ESI-TOF-Mass Analysis

After Vis and CD measurements, all samples were frozen and stored at −20 °C until mass analysis. The frozen samples were quickly melted under r.t., and the sample solutions were filtered using a cartridge filter (cellulose acetate, pore size: 0.45 μm, Toyo Roshi). These sample solutions were injected directly by syringe pump (flow rate: 180 µL/hr, KDS-100-CE (KD Scientific Inc., Holliston, MA, USA) without adding any flow-solvent. Electrospray ionization-time of flight mass spectrometry (ESI-TOF-Mass) analysis was performed using a micrOTOF-QII mass spectrometer (Bruker, Billerica, MA, USA) and analyzed using the included software. Mass measurement was performed over the mass range (*m*/*z*) from 100 to 3500. HR-MS was recorded by micrOTOF-QII, and calibration was performed with TuneMIX (Agilent Technologies, Santa Clara, CA, USA). The collision energy of the MS/MS analysis was set at 20 eV (positive detection) or 30 eV (negative detection).

## 4. Conclusions

In conclusion, we obtained the hydrangea blue-complex by mixing Dp3G (**1**), 5CQ (**1**) or 5*p*CQ (**3**) and Al^3+^ in very low-concentration buffer solutions at pH 4.0. By ESI-TOF-Mass analysis, we detected the molecular ion peak, which is attributable to the ratio of **1**, **2** or **3** and Al^3+^ of 1:1:1. Thus, we determined the structure of the complex responsible for the sepal blue coloration to be Dp3G-Al^3+^-5-*O*-acylquinic acid.

## Figures and Tables

**Table 1:** λ_vismax_ and absorbance of reproduced solutions. Dp3G (**1**, 0.1 mM), Al^3+^ (0.1 mM, 1 eq.) in 2 mM buffer solution.

Co-Pigment	pH	λ_max_ (A_max_)			
		0 eq.	1 eq.	2 eq.	3 eq.
5CQ (**2**)		566 (0.77)	576 (1.28)	579 (1.49)	581 (1.51)
5*p*CQ (**3**)	4.0		578 (1.56)	580 (1.94)	582 (1.99)
3CQ (**4**)			569 (0.91)	569 (0.64)	569 (0.61)
5CQ (**2**)		525 (0.55)	543 (0.67)	567 (0.74)	576 (0.96)
5*p*CQ (**3**)	3.2		569 (0.73)	575 (0.95)	578 (1.22)
3CQ (**4**)			531 (0.54)	530 (0.55)	531 (0.58)

## Supplementary Materials

The following are available online: Figures S1–S6 and Table S1.
