# Supplementary material for: Direct Observation of Hydrangea Blue-Complex Composed of 3-O-Glucosyldelphinidin, Al3+ and 5-O-Acylquinic Acid by ESI-Mass Spectrometry
Source: Molecules. 2018 Jun 12;23(6):1424. doi: 10.3390/molecules23061424 (PMC6100629; doi:10.3390/molecules23061424)
Supplement: Supplementary file 1 [file molecules-23-01424-s001.pdf]

Direct Observation of Hydrangea Blue-Complex Composed of 3-*O*-Glucosyldelphinidin, Al^{3+} and 5-*O*-Acylquinic Acid by ESI-Mass Spectrometry

Takaaki Ito [1], Kin-ichi Oyama [2] and Kumi Yoshida [3,*]

[1] Graduate School of Information Sciences, Nagoya University, Chikusa, Nagoya 464-8601, Japan; ito.takaaki@b.mbox.nagoya-u.ac.jp
[2] Research Institute for Materials Science, Nagoya University, Chikusa, Nagoya 464-8602, Japan; oyama@cic.nagoya-u.ac.jp
[3] Graduate School of Informatics, Nagoya University, Chikusa, Nagoya 464-8601, Japan
* Correspondence: yoshidak@i.nagoya-u.ac.jp; Tel.: +81-052-789-5638

1. Supplemental figures

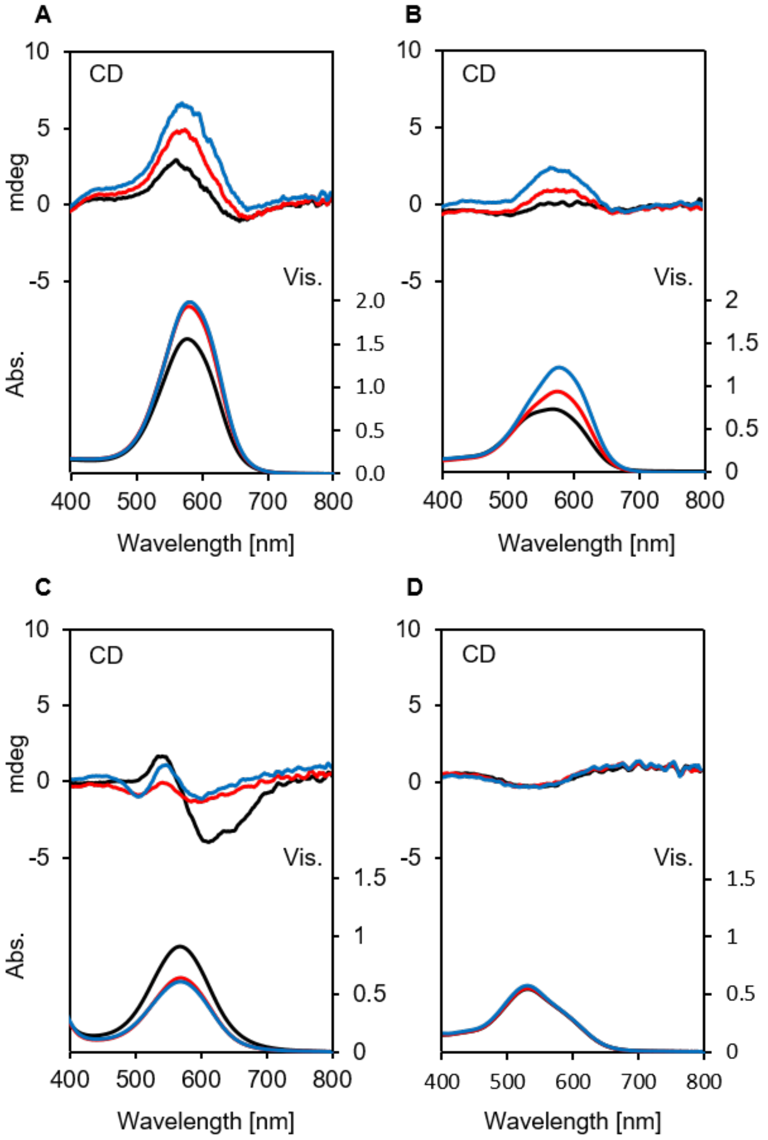

Figure S1. Visible and CD spectra of reproduced solutions by mixing **1** (Dp3G, 0.1 mM) and Al^{3+} (1 eq.) with 1-3 eq. of co-pigment, 5*p*CQ (**3**), or 3CQ (**4**) in buffered solutions of 2 mM. —: 1 eq., —: 2 eq., —: 3 eq. (**A**) With 5*p*CQ (**3**) at pH 4.0. (**B**) With 5*p*CQ (**3**) at pH 3.2. (**C**) With 3CQ at pH 4.0. (**D**) With 3CQ at pH 3.2.

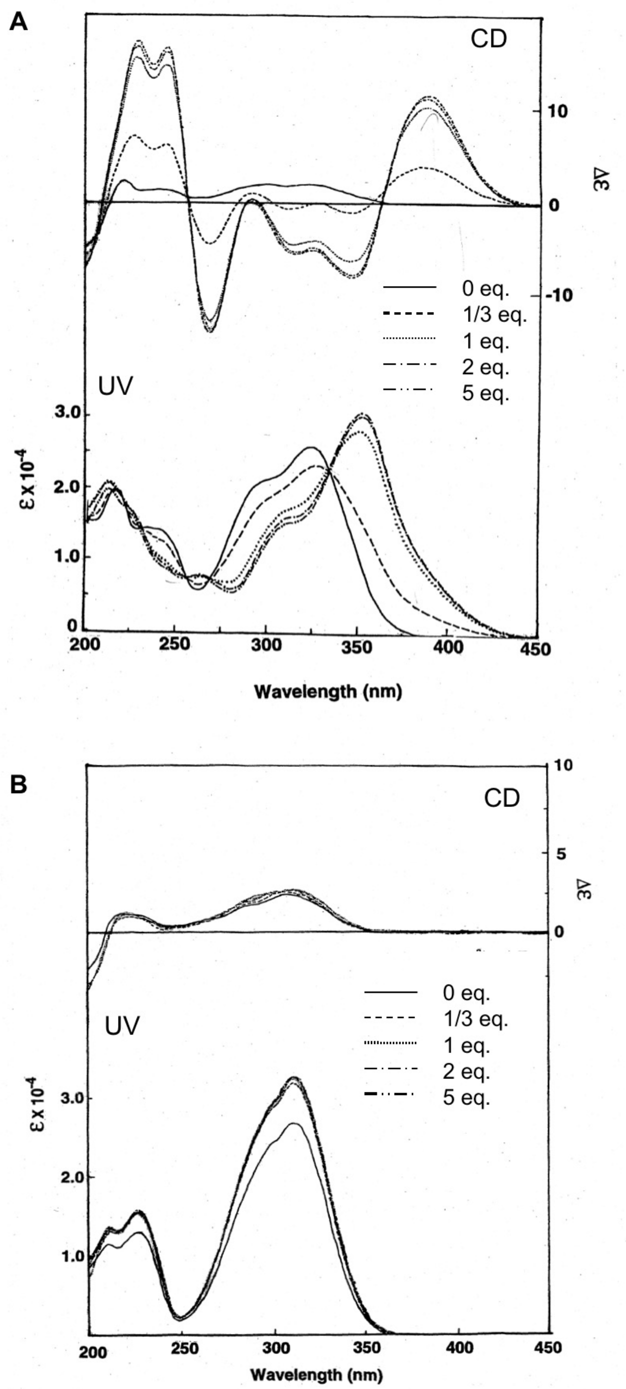

Figure S2. UV and CD spectra of co-pigments (5 mM) with Al^{3+} (0-5 eq.) at pH 4.5. (**A**) 5CQ (**2**), (**B**) 5*p*CQ (**3**).

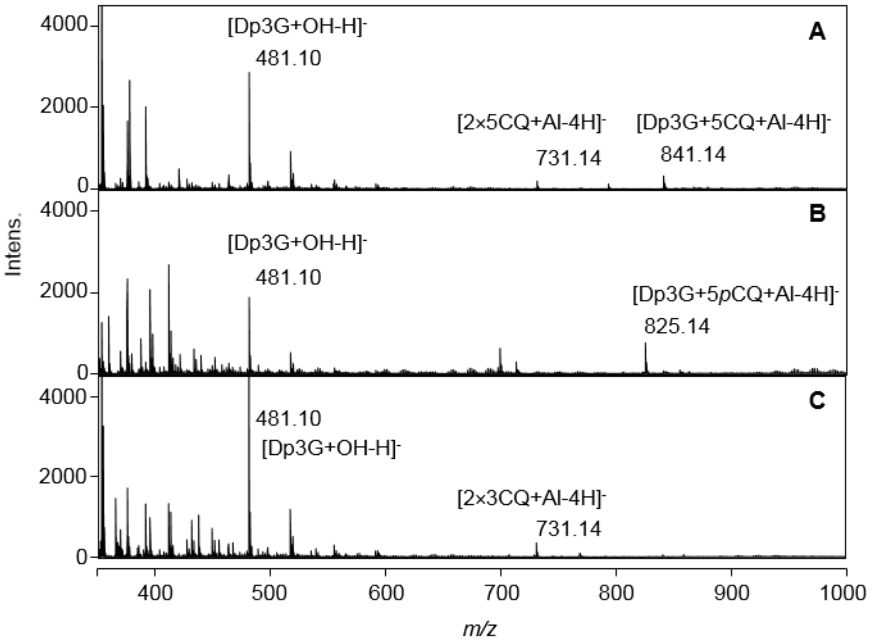

Figure S3. Negative detection ESI-TOF-MS spectra of reproduced solutions by mixing **1** (Dp3G, 0.1 mM) and Al^{3+} (1 eq.) with 2 eq. of co-pigment, 5CQ (**2**), 5*p*CQ (**3**), or 3CQ (**4**) in buffered solutions at pH 4.0 (2 mM). (**A**) 5CQ (**2**). (**B**) 5*p*CQ (**3**). (**C**) 3CQ (**4**).

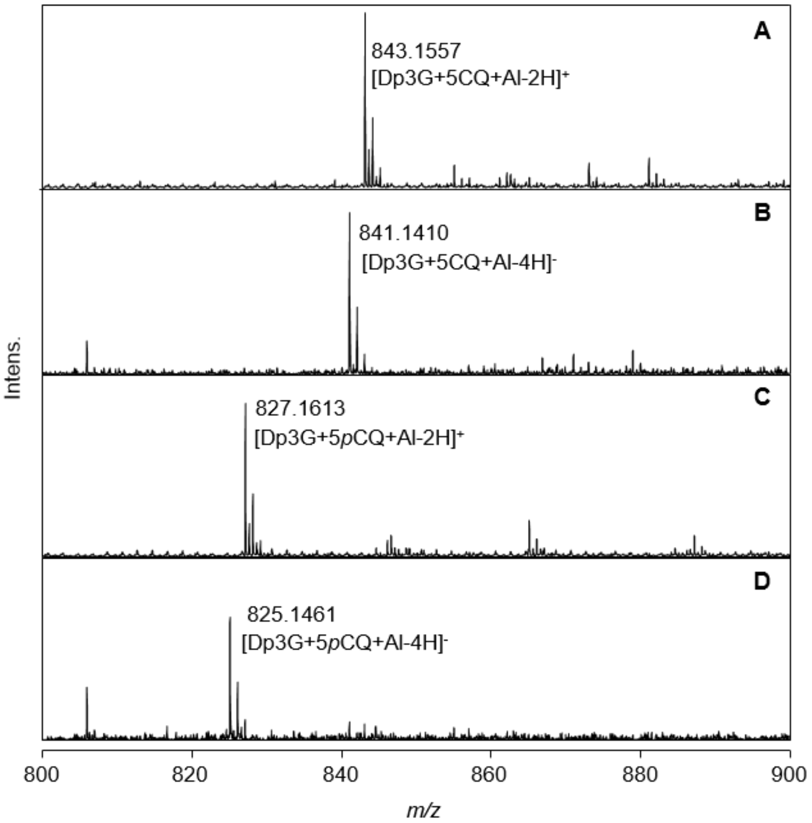

Figure S4. High resolution ESI-TOF-MS spectra of reproduced solutions by mixing **1** (Dp3G, 0.1 mM) and Al^{3+} (1 eq.) with 2 eq. of co-pigment, 5CQ (**2**), 5*p*CQ (**3**) in buffered solutions at pH 4.0. (**A**) Positive mode, 5CQ (**2**), calcd for $C_{37}H_{36}O_{21}Al$ $[M+H]^+$ 843.1559, found 843.1557. (**B**) Negative mode, 5CQ (**2**), calcd for $C_{37}H_{34}O_{21}Al$ $[M-H]^-$ 841.1413, found 841.1410. (**C**) Positive mode, 5*p*CQ (**3**), calcd for $C_{37}H_{36}O_{20}$ Al $[M+H]^+$ 827.1610, found 827.1613. (**D**) Negative mode, 5*p*CQ (**3**), calcd for $C_{37}H_{34}O_{20}Al$ $[M-H]^-$ 825.1464, found 825.1461.

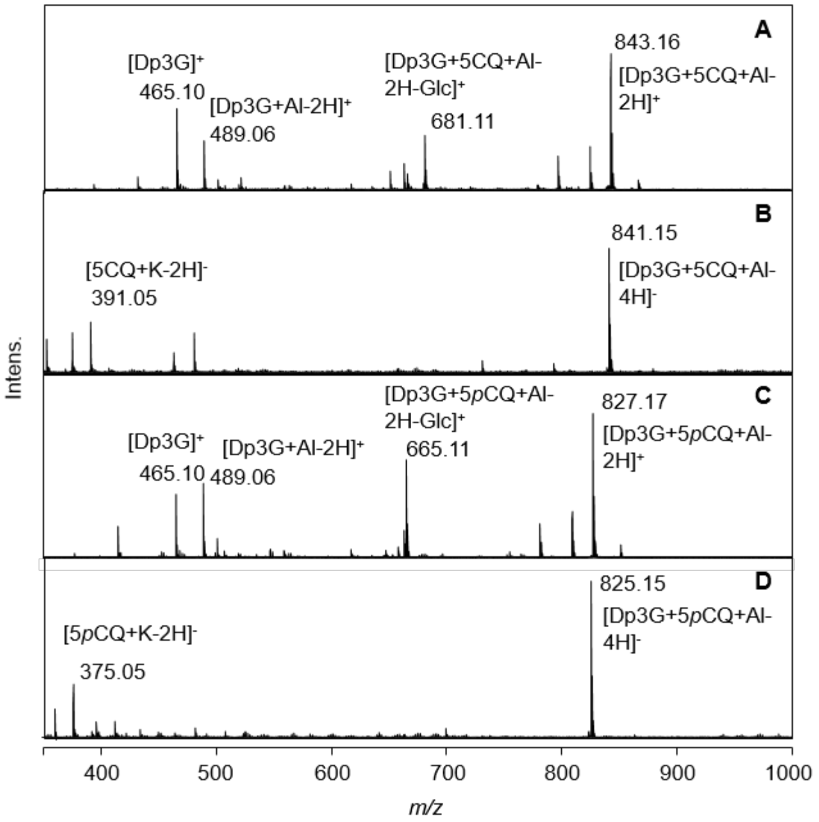

Figure S5. ESI-TOF-MS/MS spectra of the blue complex reproduced by mixing **1** (Dp3G, 0.1 mM) and Al^{3+} (1 eq.) with 2 eq. of co-pigment, 5CQ (**2**) or 5*p*CQ (**3**) in buffered solutions at pH 4.0 (2 mM). (A) Dp3G-Al-5CQ (positive mode, collision energy 20 eV). (B) Dp3G-Al-5CQ (negative mode, collision energy 30 eV). (C) Dp3G-Al-5*p*CQ (positive mode, collision energy 20 eV). (D) Dp3G-Al-5*p*CQ (negative mode, collision energy 30 eV).

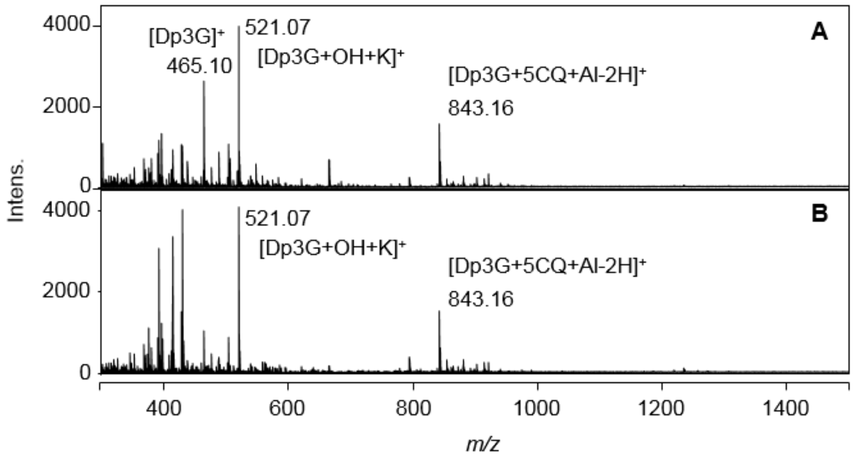

Figure S6. Positive detection ESI-TOF-MS spectra of reproduced solutions by mixing **1** (Dp3G, 0.1 mM) and Al^{3+} (1 eq.) with 1 and 3 eq. of co-pigment, 5CQ (**2**) in buffered solutions at pH 4.0 (2 mM). (**A**) 1 eq., (**B**) 3 eq.

2. Elemental analysis of blue hydrangea cell sap

Metal contents in cell sap from hydrangea sepal was performed by Inductively coupled plasma-optical emission spectrometry (ICP-OES) analysis after wet ashing. 100 μL cell sap was collected into a PTFE tube, then added 2.5 mL HNO_3 (d=1.38, for metal analysis grade, WAKO) and heated at 105 °C for 2 hours in Digi-PREP Cube (SCP Science). Next 200 μL 30% H_2O_2 (WAKO) was added and heated at 160 °C for 16 hours. After cooling the solution was messed up to 20 mL and filtered with cellulose acetate filter (0.45 μm, TOYO Roshi). ICP-OES analysis was performed with Vista-PRO (VARIAN). The concentration of each metal was determined with calibration curves from standard solution (ICP multi-element standard solution IV, Merck). The result is summarized in Table S1.

Table S1. Metal contents in cell sap from blue hydrangea sepal.

Metal	Conc. [mM]
Na	0.93
Mg	5.2
Al	2.9
K	30
Ca	2.4
